# Temporal GWAS and QTL mapping of UAV-derived digital canopy height reveals stage-specific genetic effects in bread wheat

**DOI:** 10.1016/j.plaphe.2026.100279

**Published:** 2026-09-03

**Authors:** Fabio Fania, Patrizio Spadanuda, Damiano Puglisi, Giuseppina Angione, Ida Colella, Ivano Pecorella, Salvatore Esposito, Pasquale De Vita

**Affiliations:** a CREA Research Centre for Cereal and Industrial Crops, CREA-CI, Foggia, Italy; b Department of Agriculture, Food, Natural Resources and Engineering (DAFNE), University of Foggia, Foggia, Italy; c Department of Agriculture and Forest Sciences, University of Tuscia, Viterbo, Italy; d CNR-IBBR, National Research Council of Italy, Institute of Biosciences and Bioresources, Portici, NA, Italy

**Keywords:** UAV phenotyping, Digital canopy height, Tillering plateau, *Rht*, Adaptation genes

## Abstract

Dissecting the temporal genetic architecture of crop growth requires phenotyping platforms that combine high throughput with fine temporal resolution. Here, we combined multi-season RGB-UAV phenotyping with temporal GWAS and QTL mapping to resolve stage-specific genetic effects on canopy height in bread wheat. An elite panel of 174 cultivars and a recombinant inbred line population of 162 lines were monitored across three growing seasons (2021-2024) in southern Italy. Digital canopy height was extracted from plot-level 3D point clouds at multiple percentile levels and validated against ground measurements (R^2^ = 0.89-0.98; RMSE = 0.07-0.20 m). Temporal modelling of fitted height trajectories generated both whole-height and growth-rate traits; growth-rate associations consistently preceded whole-height associations by 100-200 growing degree days (GDD), capturing the onset of genetic effects on elongation earlier than cumulative height. An apparent tillering plateau separated two phases of genetic control: an early phase linked to vernalization (*Vrn*) and photoperiod (*Ppd*) loci and a later phase linked to gibberellin-related dwarfing genes (*Rht*), a sequence consistent with published expression profiles. Recurrent loci converged on five chromosomal regions, and candidate-gene prioritization highlighted a small set of regulators, including DELLA and TB1-like genes. Overall, temporal UAV phenotyping, and especially the contrast between whole-height and growth-rate traits, resolves when successive developmental loci become detectable on the canopy, providing a framework to dissect the dynamic genetic control of plant height.

## 1 Introduction

Bread wheat (*Triticum aestivum* L.) is the third most widely cultivated cereal crop worldwide, contributing approximately 20% of human caloric intake [1,2]. Climate change threatens wheat productivity through altered growth dynamics and developmental timing [[3], [4], [5]], underscoring the need for a detailed understanding of the genetic and environmental factors governing wheat adaptation. Among adaptive traits, canopy height and phenological timing are of paramount importance [6]. Optimizing plant height improves harvest index, lodging resistance, and the capacity for light interception and biomass accumulation [7]. However, most genetic studies of wheat height rely on static measurements taken at maturity, overlooking the differential gene activity occurring throughout development [8]. While the semi-dwarf *Rht-B1b* and *Rht-D1b* alleles on chromosomes 4B and 4D are well-characterized regulators of final height [9], numerous additional QTLs have been identified across nearly all 21 wheat chromosomes [10,11]. Importantly, adaptation genes such as *Vrn* and *Ppd* modulate growth dynamics by fine-tuning the duration of developmental phases [[12], [13], [14]], exerting pleiotropic effects not only on flowering time but also on plant stature and canopy architecture [6]. Yet the temporal effects of these genes on canopy height are difficult to detect with endpoint measurements alone.

High-throughput phenotyping (HTP) technologies, particularly drone-based remote sensing, have transformed the capacity to monitor crop canopy dynamics across large experimental fields [[15], [16], [17]]. Digital surface models (DSMs) derived from Structure-from-Motion (SfM) algorithms enable precise, non-destructive estimation of canopy height at multiple time points, providing the temporal resolution needed to probe dynamic genetic effects. Studies in sorghum [18], maize [19], and, increasingly, wheat [20,21] have demonstrated the value of this approach for the genetic dissection of growth-related traits. More recently, temporally resolved UAV phenotyping has been applied to dissect stage-specific QTLs for plant height in maize [22,23] and cotton [24], revealing that different genomic regions control height at different developmental stages and that growth-rate traits can uncover associations not detectable from endpoint measurements alone. Indeed, the temporal canopy-height trajectories can be decomposed into two complementary descriptors of genetic effects: whole height, which integrates all genetic and environmental contributions from seedling emergence to a given growth stage, and instantaneous growth rate, defined as the temporal derivative of the fitted height curve. These two descriptors capture different facets of the same genetic architecture. Whole height reflects persistent, cumulative effects but may detect associations with a temporal delay relative to the onset of gene activity; growth rate, in contrast, isolates marginal genetic contributions within narrow time windows, conferring greater sensitivity to the timing of gene activation at the cost of increased transient noise [18,25]. Their joint analysis within a temporal mapping framework therefore provides a more complete portrait of genetic control than either descriptor alone: growth rate reveals when a locus begins to act, while whole height indicates how strongly it contributes to the final phenotype. Despite this conceptual advantage, the joint use of both descriptors has rarely been implemented in temporal genetic studies of wheat canopy height.

A systematic time-series analysis, explicitly linking UAV-derived growth curves to the activation timing of specific adaptation genes, remains limited in wheat. In particular, three questions remain open: (i) whether growth-rate traits derived from temporal UAV profiles detect genetic associations earlier than whole-height traits; (ii) whether physiologically distinct developmental phases (e.g., tillering, stem elongation, heading) display qualitatively different patterns of genetic control; and (iii) whether a conserved set of loci underlies canopy height trajectories across seasons and genetic backgrounds.

To address these questions, we combined RGB-UAV phenotyping with temporal GWAS and QTL mapping in two complementary bread wheat populations evaluated over three consecutive growing seasons in southern Italy. We first validated the accuracy of UAV-derived digital canopy height against manual ground-truth measurements using a multi-percentile extraction strategy designed to distinguish the dominant canopy surface from noise-prone extremes, then exploited the temporal resolution of the validated platform to identify stage-specific genetic associations and candidate genes underlying wheat canopy height development. Finally, we compared the temporal patterns of marker-trait associations with published gene expression profiles to assess the biological coherence of the detected signals.

## 2 Materials and methods

### 2.1 Plant materials and field trials

**Fig. 1:**
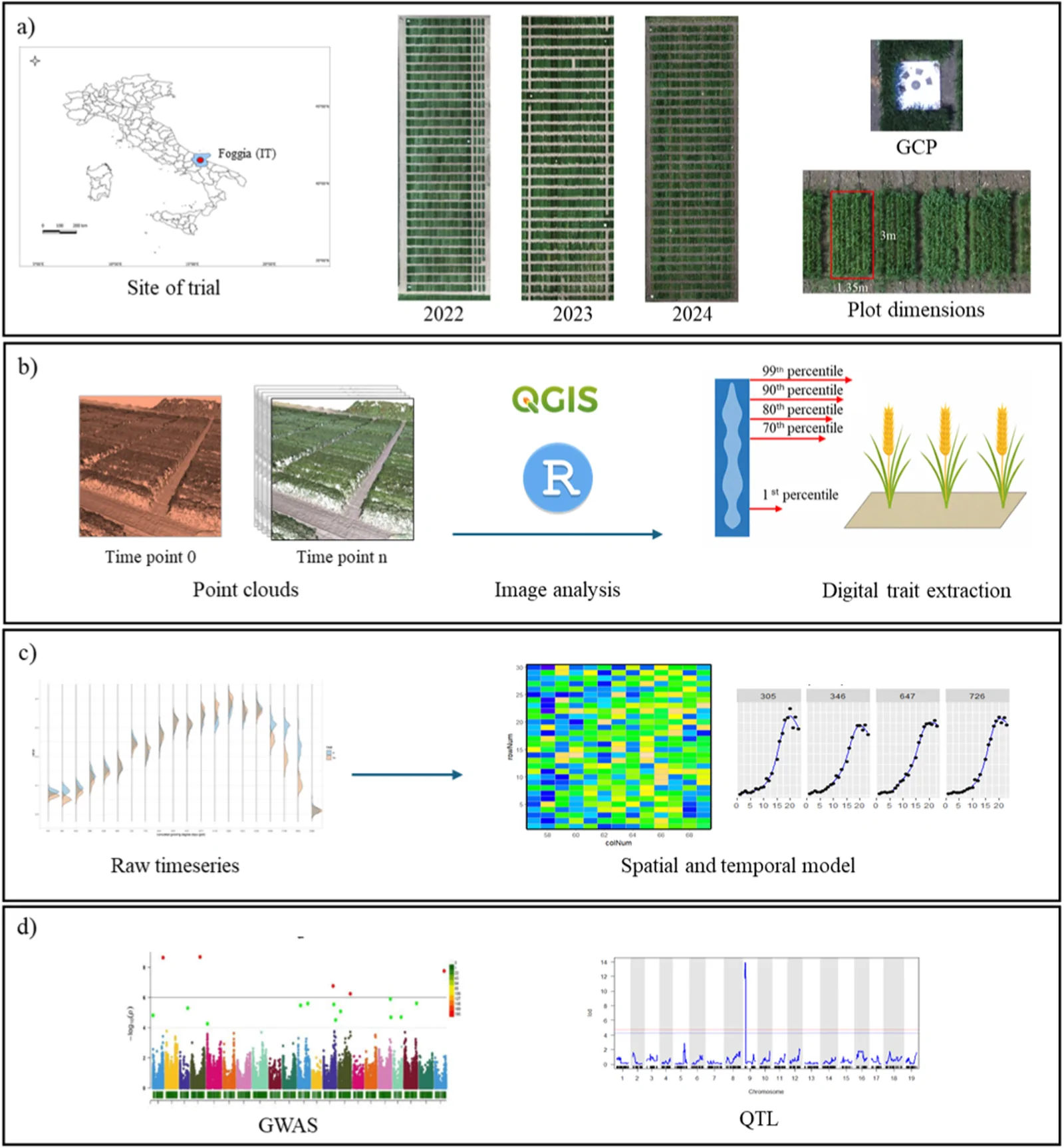
Overview of the experimental design and analysis workflow for UAV-derived digital trait extraction and downstream genetic analyses. (a) Trial site location (Foggia, Italy) and example of plot layout across the 2021–2024 seasons, including ground control points (GCPs) and plot dimensions. (b) Generation of point clouds at multiple time points and image/point-cloud processing in QGIS and R to derive digital traits; example of percentile-based extraction (1st and 99th percentiles) (c) Plot-level raw time series and subsequent spatial and temporal modeling to obtain smoothed dynamic profiles and summary matrices. (d) Identification of genomic regions associated with the target traits through GWAS and QTL analyses.

Field experiments were conducted at the Research Centre for Cereal and Industrial Crops (CREA-CI) in Foggia, Southern Italy (41°46′ N, 16°54′ E) over three consecutive growing seasons (2021-22, 2022-23, and 2023-24) (Fig. 1a).

Two distinct bread wheat populations were studied: an elite panel comprised 174 cultivars released in Italy over the past two decades, previously described by Vitale et al. (2024) [26] and a RIL population consisting of 162 F7-derived lines from the cross Lankaodali x Rebelde, previously characterized by Vitale et al. (2021) [27] (Table S1). Both populations were arranged in augmented experimental designs with two check cultivars replicated 23 times to facilitate spatial correction. Experimental units were 4 m^2^ plots (1.33 m × 3 m) sown at 350 seeds m^−2^ during the second week of December each year (20 December 2021; 10 December 2022; 12 December 2023).

**Fig. 2:**
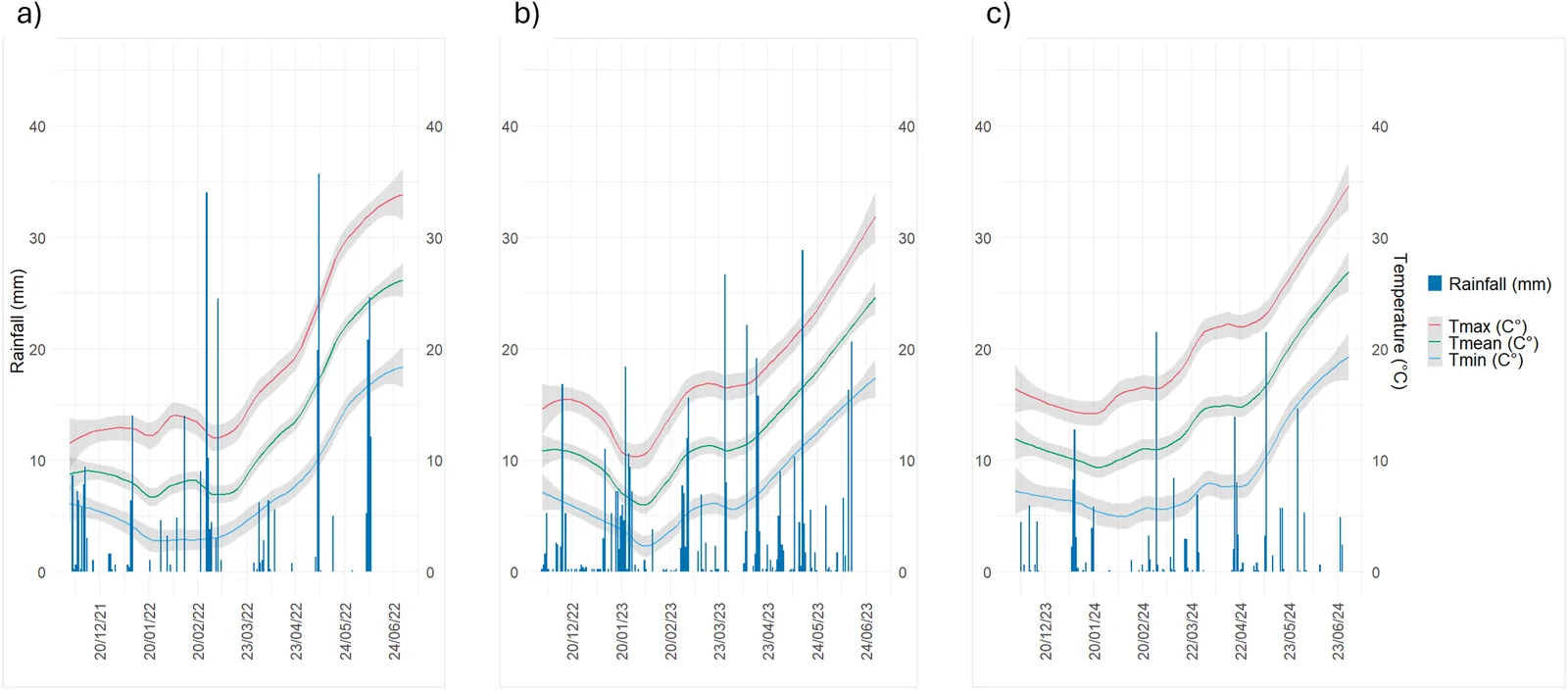
Maximum (Tmax), mean (Tmean), and minimum (Tmin) daily temperatures (°C) and precipitation (rainfall, mm) are depicted for three consecutive growing seasons: (a) 2021-22, (b) 2022-23, and (c) 2023-24.

Standard agronomic practices were applied, including nitrogen fertilization at BBCH 29 (end of tillering) and a single annual herbicide application. No fungicide treatments were applied. Daily weather data were recorded by an on-site station (Fig. 2), and growing degree days (GDD) were calculated following Baskerville and Emin (1969) [28] using the pollen package in R [29]. GDD was used as the temporal scale throughout, as it provides greater biological comparability than calendar date or days after sowing. For the phenological phase-based environmental comparison, each growing season was divided into three developmental periods using the observed phenological dates: vegetative phase, from emergence to the end of the tillering stage; reproductive phase, from stem elongation (BBCH31) to heading (BBCH59); and grain filling period, from BBCH59 onward. For each phase and season, cumulative rainfall, mean temperature, accumulated thermal time, the median and range of H^2^ for DCH70-DCH99, and the number and chromosomal distribution of non-redundant temporal MTAs/QTLs were summarized separately for the elite panel and RIL population (Table S2).

### 2.2 Ground-based canopy height measurements

Manual ground canopy height (GCH) was measured during the first growing season (2021-22), on both the elite panel and the RIL population, at seven time points spanning tillering (BBCH 21) to flowering time (BBCH 69). To improve measurement standardization, a lightweight cotton cloth was positioned horizontally across each plot canopy, and height was recorded through five apertures along the cloth's diagonals using a rigid measuring tape (±0.5 cm accuracy). Plot-level GCH was computed as the mean of the five readings. It should be noted that this method introduces a systematic measurement bias that must be acknowledged when interpreting the validation results. When the cloth is laid horizontally across the canopy, it does not rest on the tallest individual structures - such as the apices of dominant tillers, flag-leaf tips or isolated awns, but rather settles on the load-bearing layer formed by the collective upper canopy surface, i.e. the height at which the canopy provides sufficient mechanical support to sustain the cloth without appreciable compression, effectively filtering out the sparse uppermost elements that define the true maximum height. From a distributional standpoint, this load-bearing surface is expected to correspond most closely to upper-intermediate percentiles of the digital canopy height distribution, specifically the 80th to 90th percentiles of canopy height. Therefore, the GCH data will be interpreted as a reference for the dominant canopy surface height rather than for the whole canopy maximum.

To provide an independent reference against conventional maximum plant height, an additional validation panel of 46 bread wheat genotypes, the genetically divergent lines characterized for plant height by Esposito et al. [30] using the reverse BSA-QTLseq approach, was evaluated during the 2022-2023 growing season in a separate, parallel field experiment conducted under irrigated and water-stressed conditions. In this experiment, plant height was measured directly in the field using a rigid measuring tape at seven time points, based on five measurements per plot and without the cloth procedure, and was used exclusively as an independent reference for validating UAV-derived canopy-height percentiles against field-measured plant height.

Phenological phases, including seedling emergence (BBCH 10), stem elongation onset (BBCH 31), and heading date (HD, BBCH 59), were recorded across all three growing seasons when at least 50% of plants in a plot reached the corresponding stage. Dates were converted into GDD for downstream analyses (Table S3).

### 2.3 UAV acquisition and digital canopy height extraction

Ground control points (GCPs) were established each season at a density of 4-5 per hectare, exceeding the commonly recommended range of 0.5-1 GCP ha^−1^ for UAV photogrammetric surveys [31]. Each GCP covered an area of 1 m^2^ and was georeferenced with a high-precision Trimble RTK system providing centimetre-level accuracy (typically ± 1-2 cm in the horizontal plane and ± 2-3 cm in the vertical plane under fixed-RTK correction). This high GCP density, combined with centimetre-level RTK positioning, ensured reliable altimetric accuracy for digital canopy height extraction. UAV flights were conducted throughout each season from the day after sowing (time zero) until harvest (Table S4), using a DJI Phantom 4 Pro equipped with an RGB camera (20 MP effective resolution, 1-inch CMOS sensor, adjustable aperture f/2.8-f/11). Flights were performed at 30 m altitude, 4 m/s speed, with 80% front and 75% side image overlap. All flights were conducted between 12:00 and 14:00 on clear-sky days (cloud cover < 10%, wind < 10 m/s) to minimize shadow effects and ensure consistent illumination geometry across time points.

Orthomosaics and 3D point clouds were generated using Pix4D Mapper (v4.7.5) via the Structure-from-Motion algorithm. Point clouds were processed using a custom R pipeline integrating FIELDImageR [32], terra [33] and lidR [34]. Plot boundaries were defined using georeferenced shapefiles derived from the experimental field layout and internally buffered by 0.5 m in QGIS to minimize edge effects and contamination from adjacent plots. Only points located within the buffered plot extraction areas were retained for plot-level analysis. The original point clouds had an average density of 1900 points m^−2^, with a range of 1500-2300 points m^−2^, and were spatially homogenized to a target density of 1500 points m^−2^ using a 50-m grid; the resulting effective density after decimation was 1380 points m^−2^. Point heights were normalized by direct subtraction of a digital surface model generated from the early-season bare-soil flight acquired the day after the sowing, using the same coordinate reference system as the point clouds. Normalized points outside the biologically plausible height range of 0–3 m were removed, while statistical outliers were identified using the statistical outlier removal algorithm implemented in lidR [34], with ten nearest neighbours, a multiplier of 0.9, and quantile-based thresholding. The remaining points were smoothed using a circular Gaussian moving window of 0.5 m and a standard deviation of 1 to reduce high-frequency reconstruction artifacts while preserving spatial variability among plots. Plot-level UAV-derived digital canopy height (DCH) traits were calculated directly from the normalized, denoised, and smoothed point-height distributions, without rasterization or spatial interpolation. The 40th, 50th, 60th, 70th, 80th, 90th, and 99th percentiles were extracted for each plot and are hereafter denoted DCH40, DCH50, DCH60, DCH70, DCH80, DCH90, and DCH99, respectively. Throughout, the DCH40-DCH60, DCH70-DCH90, and DCH99 percentiles are referred to as the lower, intermediate, and uppermost percentiles, respectively. Of these seven extracted percentiles, the four upper percentiles (DCH70, DCH80, DCH90, and DCH99) were carried forward to the temporal GWAS and QTL analyses, whereas the lower percentiles (DCH40-DCH60) were used only to validate the UAV estimates against the ground-based measurements. The normalized DCH model does not yield a single height value per plot but rather a continuous distribution of elevation values reflecting the three-dimensional heterogeneity of the canopy surface. Different summary statistics extracted from this distribution sample different vertical layers and are therefore expected to differ in their noise level, biological content, and genetic informativeness.

Plot-level DCH was extracted at seven percentile levels (DCH40-DCH99) from the normalized canopy height model (Fig. 1b). These percentile-based metrics represent the vertical distance from the soil surface to the corresponding canopy layer, herein termed “whole height”.

This multi-percentile extraction strategy was adopted to systematically evaluate the effect of vertical sampling position on trait quality and mapping power and was motivated by three considerations. First, noise suppression: the uppermost percentile (DCH99) captures the tallest structures within a plot - including individual awns, flag-leaf tips, and occasional point-cloud reconstruction artifacts - and are therefore inherently more variable and less representative of the bulk canopy; conversely, percentiles below approximately the 50th increasingly incorporate soil-exposed regions and inter-row gaps. Intermediate percentiles (DCH70-DCH90) are expected to represent a biological optimum that describes the dominant canopy surface while filtering both upper-canopy noise and lower-canopy soil contamination. Second, biological stratification: because different canopy layers are shaped by partially distinct genetic determinants - spike length and awn presence for the uppermost layer, internode elongation and leaf architecture for the bulk surface - the multi-percentile design allows testing whether the QTL/MTA landscape varies as a function of the vertical layer sampled. Third, robustness of genetic inferences: associations detected consistently across multiple percentile levels are more likely to reflect genuine genetic effects than signals observed at a single level, providing an internal replication axis that complements replication across years and populations. Based on these considerations, we formulated three testable predictions: (a) intermediate percentiles would show higher heritability and repeatability than the DCH99 percentile; (b) different percentiles would reveal partially distinct sets of associations; and (c) the concordance between digital and ground-truth measurements would be highest for the percentiles that best approximate the load-bearing canopy surface measured by the cloth method (DCH80-DCH90).

The reproducible workflow used for point-cloud processing and plot-level DCH extraction are publicly available in a version-controlled GitHub repository (https://github.com/devitagroup/Temporal-association-studies-on-digital-canopy-height.git) and the example data are available on Zenodo (https://doi.org/10.5281/zenodo.21532537).

### 2.4 Statistical analysis

All statistical analyses were performed in R v4.2 [35]. Spatial field heterogeneity was corrected using two-dimensional P-spline models implemented in SpATS [36], yielding best linear unbiased predictions (BLUPs) for all traits separately for each growing season. Broad-sense heritability (H^2^) was computed following the effective dimensions approach described by Rodríguez-Álvarez et al. (2018) [36].

Spatially corrected BLUPs were used as input for temporal modelling with P-spline curves implemented in StatGenHTP [37], enabling the integration of GCH and DCH within a unified temporal framework (Fig. 1c). The knots parameter was fixed at 12, while the smoothing parameter was estimated by restricted maximum likelihood. This setting was selected through a sensitivity analysis comparing values of 6, 12, 18, and 24. The six-knot setting produced comparatively constrained trajectories, whereas the 18- and 24-knot settings introduced local oscillations and implausible peaks; the 12-knot setting provided the best balance between temporal flexibility and fitting stability. Growth-rate traits were calculated as finite differences between consecutive fitted canopy-height values on the daily prediction grid. Negative differences caused by minor local fluctuations in the fitted trajectories were set to zero. The resulting values therefore represent mean daily growth increments rather than instantaneous analytical derivatives. As an additional methodological check, the finite-difference estimates were compared with the first derivatives returned by the fitted P-splines across genotypes and time points. Growth rates were expressed in m GDD^−1^ and were subsequently used as traits for genetic analysis alongside the whole height values.

The agreement between DCH and GCH was assessed using the coefficient of determination (R^2^) and root mean square error (RMSE). These metrics were computed across all time points within the 2021-22 growing season, in which both measurement methods were applied (seven dates spanning BBCH 21 to BBCH 69).

### 2.5 Genotypic data (SNPs) and association mapping

Genomic DNA was extracted from young leaf tissue. The elite panel was genotyped using the Illumina Wheat 25K SNP array, whereas the RIL population was genotyped using the Illumina Wheat 20K array. SNP physical positions were assigned to the Chinese Spring RefSeq v2.1 reference genome [38]. Markers were filtered for call rate > 95%, minor allele frequency (MAF) > 0.05, and complete genotypic data (no missing values) [39]. After filtering, 9805 SNPs were retained for the elite panel and 3746 for the RIL population. Filtering was performed using TASSEL v5.0 [40].

Genome-wide association studies (GWAS) were performed on the elite panel for all temporal traits (whole height and growth rate at each fitted time point for the four upper percentiles (DCH70, DCH80, DCH90 and DCH99)) as well as for phenological traits (GS30 and GS59), using the BLINK model [41] implemented in GAPIT v3 [42]. Population structure was assessed a priori using STRUCTURE v2.3.4 [43] with K = 2-7, four independent runs per K, 10,000 burn-in iterations, and 20,000 MCMC iterations. The optimal number of subpopulations was determined using the ΔK method implemented in CLUMPAK [44] and was estimated at K = 3 (Fig. S1). The resulting Q-matrix of membership coefficients was included as a covariate in the BLINK model.

QTL mapping in the RIL population was performed via genome-wide composite interval mapping (gCIM) using the R/qtl package [45]. Redundant co-segregating markers exhibiting identical segregation patterns were binned to remove non-informative loci. The analysis employed a genome scan step size of 1 cM and the Kosambi mapping function. LOD significance thresholds were determined by 1000 trait-specific permutation tests at the genome-wide α = 0.05 level, and QTL additive and dominance effects were estimated using empirical Bayesian methods. QTLs were named following the recommended rules for gene symbolization in wheat (available online at https://wheat.pw.usda.gov/ggpages/wgc/98/Intro.htm, accessed 2 April 2026).

### 2.6 Temporal resolution and the 50-GDD minimum detection window

A central parameter of the temporal GWAS/QTL analysis is the minimum duration over which a marker-trait association must persist to be considered biologically meaningful rather than a transient statistical fluctuation. We adopted a minimum window of 50-GDD. Under the field conditions of our trials (mean daily temperature during active growth: approximately 10-18°C; base temperature: 0°C), 50-GDD corresponds to approximately 3-7 calendar days, potentially merging associations arising from distinct genetic programs. The 50-GDD threshold thus represents a compromise between temporal resolution and statistical stability, targeting the temporal scale at which discrete developmental events are known to occur in wheat. A comparable temporal resolution was independently adopted by Sweet et al. [23], who performed GWAS on plant height and growth rate at 50-GDD intervals in a diverse maize panel, providing cross-species support for this window as a biologically meaningful threshold for temporal genetic mapping in cereals.

Because GWAS and QTL analyses were performed independently at each fitted time point along the P-spline curve (spaced at 1-GDD intervals), single-time-point associations can arise by chance due to multiple testing, local instabilities in the spline fit, or minor fluctuations in trait variance. Thus, persistence was maintained for 50 consecutive tests, each conducted with slightly different phenotypic values. This substantially reduced the rate of false positives without explicit corrections at time points, as the likelihood of a spurious association persisting for more than 50 consecutive tests was extremely low. To evaluate robustness to the choice of window size, the temporal association analysis was repeated using three thresholds: 25 GDD (approximately 2-4 days), 50 GDD (reference), and 100 GDD (approximately 6-12 days). Outcomes were compared in terms of (a) number of retained associations, (b) chromosomal distribution of retained loci, and (c) temporal boundaries of association windows. All results in the main text are based on the 50-GDD threshold. The sensitivity analysis is provided in Supplementary Figs. S2 and S3.

### 2.7 Candidate gene analysis

Candidate genes were identified within ± 2 Mb flanking the peak marker of each significant MTA and within the physical intervals corresponding to each detected QTL region. The ± 2 Mb window was defined on the basis of the genome-wide linkage disequilibrium (LD) decay estimated for the same elite panel by Habib et al. (under review), where mean LD (r^2^) declined below 0.2 at ∼2 Mb (half-decay distance 2.12 Mb). Because LD decays more slowly in the biparental RIL population than in the elite panel, this window is conservative for the RIL. Gene models and their functional annotations were retrieved from the wheat RefSeq v2.1 genome assembly [38] using bedtools intersect [46]. The initial candidate set was subsequently prioritized using the QTG-Miner tool (https://wheat.cau.edu.cn/wGRN/w9/) within the wGRN platform [47], which ranks genes based on co-expression network connectivity, functional annotation enrichment, and known trait associations. Expression data were retrieved from the Wheat Expression Browser (expVIP) [48,49] to support functional interpretation. Prioritized candidates were further evaluated for stage-dependent transcriptional modulation - by querying expression levels across the 15 tissue-developmental stage combinations available in the wheat expression atlas - to identify genes whose transcriptional dynamics were most consistent with the temporal association patterns observed in our GWAS/QTL analysis and that were therefore most likely to underlie time-specific genetic effects on canopy height.

## 3 Results

### 3.1 UAV-derived canopy height faithfully reproduces ground-truth measurements

Ground canopy height increased steadily across the 2021-22 season in both populations (Table 1). In the elite panel, mean GCH rose from 0.13 m (4 March) to 0.68 m (29 April), with standard deviation increasing from 0.03 to 0.08 m and H^2^ ranging from 0.82 to 0.93. In the RIL population, GCH increased from 0.14 m to 0.76 m, with heritability ranging from 0.72 to 0.98 and markedly greater phenotypic dispersion at later stages (SD rising from 0.03 to 0.13 m), consistent with the segregating nature of this population.

**Table 1:** Summary statistics of ground canopy height (GCH) recorded across the growing season.

Population	Year	Day (dd/mm/yy)	Trait	Mean	Standard deviation	Min	Max	Heritability
elite_panel	2022	04/03/22	GCH	0.13	0.03	0.04	0.23	0.83
elite_panel	2022	14/03/22	GCH	0.17	0.04	0.1	0.29	0.90
elite_panel	2022	21/03/22	GCH	0.23	0.05	0.15	0.35	0.85
elite_panel	2022	01/04/22	GCH	0.35	0.05	0.25	0.46	0.82
elite_panel	2022	15/04/22	GCH	0.51	0.06	0.4	0.68	0.93
elite_panel	2022	21/04/22	GCH	0.59	0.06	0.45	0.78	0.84
elite_panel	2022	29/04/22	GCH	0.68	0.08	0.52	0.92	0.93
RIL	2022	04/03/22	GCH	0.14	0.03	0.07	0.2	0.72
RIL	2022	14/03/22	GCH	0.17	0.03	0.1	0.24	0.84
RIL	2022	21/03/22	GCH	0.23	0.04	0.15	0.34	0.81
RIL	2022	01/04/22	GCH	0.36	0.05	0.25	0.49	0.80
RIL	2022	15/04/22	GCH	0.54	0.07	0.36	0.72	0.96
RIL	2022	21/04/22	GCH	0.64	0.1	0.38	0.84	0.93
RIL	2022	29/04/22	GCH	0.76	0.13	0.4	1.02	0.98

Digital canopy height showed consistent seasonal dynamics across all three seasons and both populations (Table S5). Mean values increased from winter baselines of approximately 0.01-0.08 m to late-spring plateaus of 0.6-0.8 m. Heritability estimates were generally low during mid-winter but increased markedly from March to May. Consistent with prediction (a) formulated in Section 2.3, intermediate percentiles (DCH70-DCH90) exhibited higher heritability than the uppermost percentile (DCH99) throughout the active growth phase (H^2^ ≈ 0.70-0.95 for DCH70-DCH90 vs. H^2^ < 0.60 for DCH99 in 2022; Table S5), confirming that they provide more temporally stable descriptors of canopy development. The DCH99 percentile consistently showed the lowest repeatability, particularly during early developmental stages, likely because it captures stochastic canopy elements (i.e., apices of dominant tillers, reconstruction artifacts) rather than the dominant canopy surface.

**Fig. 3:**
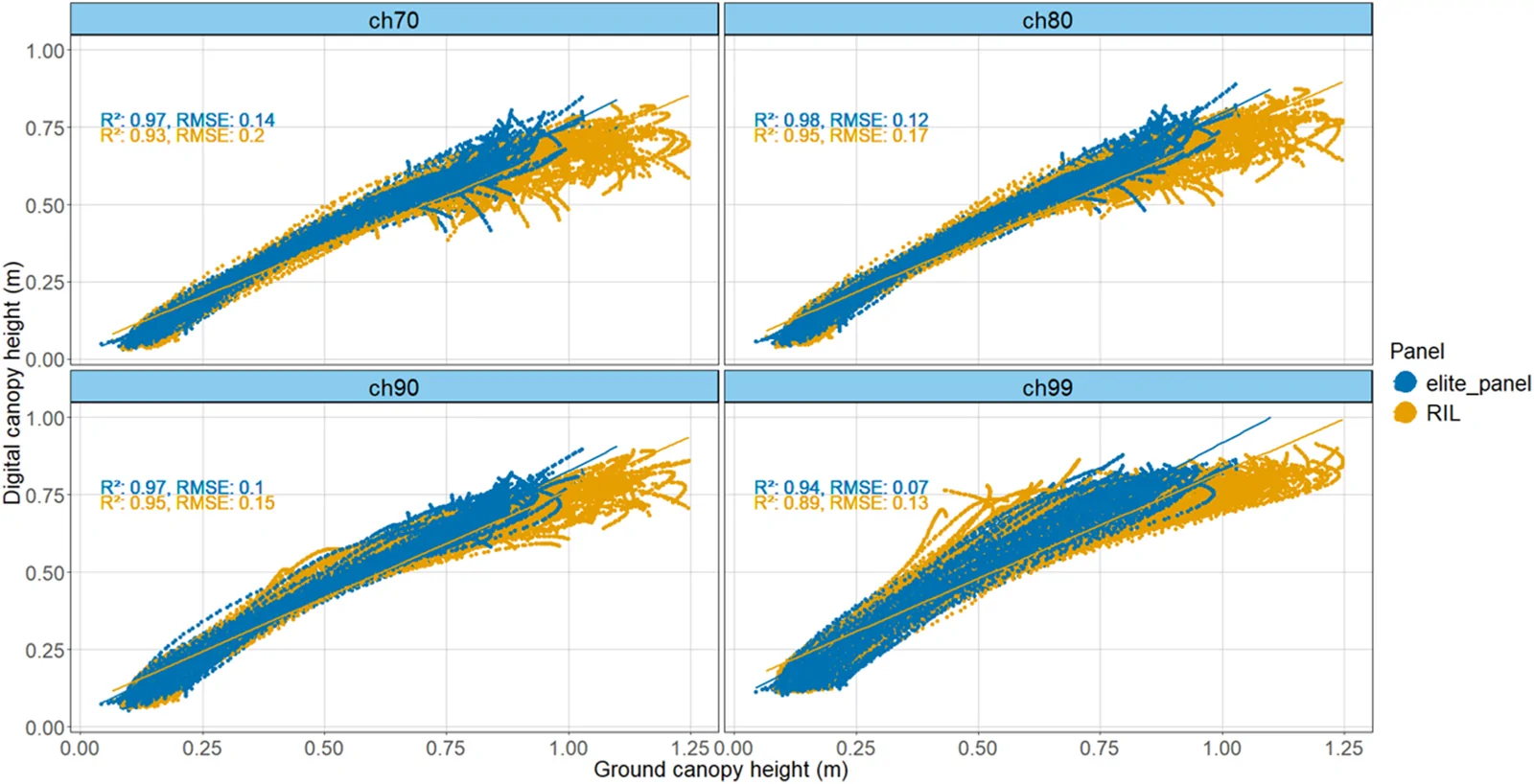
Relationship between ground canopy height (x-axis) and digital canopy height (y-axis) derived from UAV imagery for the four upper percentiles (DCH70, DCH80, DCH90, and DCH99) during the 2021-2022 growing season; agreement for all seven extracted percentiles (DCH40-DCH99) is reported in Table S6. Data points represent two study populations: elite panel (blue) and RIL population (orange). Linear regression lines are shown for each trait-population combination, along with the corresponding coefficient of determination (R^2^) and Root Mean Square Error (RMSE) values.

Validation against GCH during the 2021-22 season confirmed strong agreement across all percentile levels (Fig. 3; Table S6). R^2^ values ranged from 0.89 to 0.95 in the elite panel and from 0.94 to 0.98 in the RIL population. RMSE ranged from 0.07 to 0.20 m, with higher percentiles generally showing lower error. In agreement with prediction (c), the best correspondence with GCH was observed for DCH80 and DCH90 percentiles in both populations (R^2^ = 0.95-0.98; RMSE = 0.10-0.15 m), confirming that these percentiles most faithfully approximate the load-bearing canopy surface measured by the cloth method. Conversely, the DCH99 percentile showed systematically higher values than GCH, consistent with its sensitivity to canopy elements protruding above the bulk canopy surface. The lower percentiles (DCH40-DCH60) underestimated GCH, reflecting the inclusion of inter-row gaps and soil-exposed regions in the lower canopy layers. These results validate the multi-percentile extraction strategy and establish DCH80-DCH90 as the digital metrics most directly comparable to field-based height measurements.

The slightly higher RMSE observed in the RIL population reflects its greater phenotypic variability rather than reduced measurement quality, as the R^2^ values were in fact higher than those of the elite panel, indicating that the UAV pipeline captured a larger fraction of the genetic variance present in this segregating material.

The accuracy of UAV-derived canopy height was further evaluated using an independent panel grown in a separate field experiment, in which maximum plant height was measured directly in the field using a conventional ruler. Across irrigated and water-stressed conditions, all canopy-height percentiles showed high agreement with field measurements, with R^2^ values ranging from 0.926 to 0.957 and RMSE values ranging from 0.09 to 0.18 m (Table S7).

The robustness of the temporal modelling procedure was further evaluated through a sensitivity analysis comparing P-spline fits obtained with 6, 12, 18, and 24 knots (Fig. S5). The six-knot setting produced stable but comparatively constrained trajectories, whereas the 18- and 24-knot settings introduced local oscillations and occasional biologically implausible peaks. The 12-knot setting provided the best compromise between flexibility and stability and was therefore retained for subsequent analyses. Daily finite-difference growth increments were also highly consistent with the analytical first derivatives of the fitted P-splines, with R^2^ values of at least 0.97 across DCH percentiles and time points.

### 3.2 Growth-rate traits detect genetic associations earlier than whole-height traits

Temporal GWAS and QTL mapping across three seasons, DCH70-DCH99, and two trait types (whole height and growth rate) yielded a total of 427 non-redundant significant associations: 335 MTAs from the elite panel and 92 QTLs from the RIL population (Tables S8–S16). In the elite panel, chromosomes 5A and 7A harboured the largest numbers of MTAs (48 and 36, respectively), while in the RIL population, chromosome 1B showed the highest number of QTLs (22). The D-genome was underrepresented overall, with chromosome 6D contributing only a single QTL, consistent with the lower marker density and reduced polymorphism typically observed in the D subgenome of bread wheat (Table S10). All the QTLs, identified as described in the material and method section, are available in Table S11, while more frequent loci for digital traits were summarized in Table S12.

In addition, 14 MTAs and 12 QTLs were associated with the phenological traits (BBCH 30 and BBCH 59) across the three growing seasons (Table S13). Notably, the QTL on chromosome 5A peaking at 582 Mb co-localized with stem elongation and heading date across all three years and overlapped with the *Vrn-A1* region, a result independently confirmed by at least two MTAs at the same locus. An additional QTL on chromosome 7D (peak at 89 Mb) was associated with heading date, maturity time, and grain filling period, and mapped near an MTA for the onset of stem elongation (BBCH 30). These phenological associations provide a direct link between the canopy height dynamics analysed below and the underlying developmental timing controlled by major adaptation loci.

**Fig. 4:**
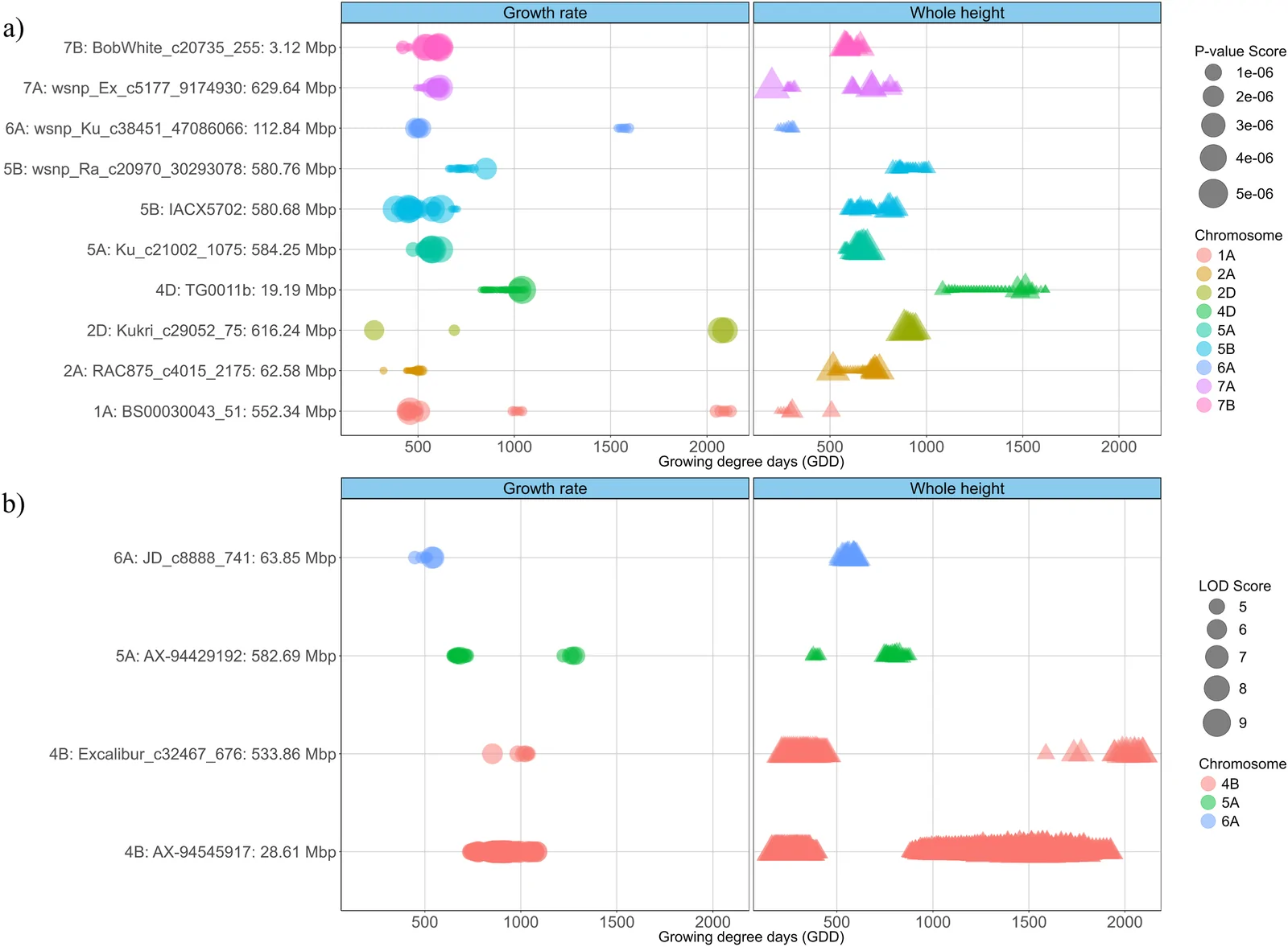
Manhattan biplots of temporal marker–trait associations (MTAs, a) and quantitative trait loci (QTLs, b) detected across three growing seasons and four canopy height percentiles (DCH70, DCH80, DCH90, and DCH99). The x-axis represents time expressed as growing degree days (GDD), and the y-axis indicates the associated markers. Colors correspond to different chromosomes, while point size reflects the significance level (–log_10_(p) for MTAs and LOD score for QTLs). Both panels are faceted by phenotype type (growth rate and whole height).

Fig. 4 provides an integrated summary of the non-redundant temporal MTAs and QTLs detected across the three growing seasons and the four DCH percentiles analysed (DCH70, DCH80, DCH90, and DCH99), with the elite panel and RIL population presented separately. Within this integrated representation, a consistent pattern emerged across populations and seasons: growth-rate associations generally appeared at earlier developmental stages than the corresponding whole-height associations. Thirty-six concordant associations (33 MTAs and 3 QTLs) were confirmed by both field and digital measurements (Table 2). Among these, growth-rate signals at a given locus preceded the corresponding whole-height signal by approximately 100-200 GDD. For example, on chromosome 4B, growth-rate MTAs at 481 and 496 emerged earlier than the corresponding whole-height signals, and *Rht-B1*, although not associated in field measurements, showed strong digital signals at the DCH70-DCH90. Similarly, on chromosome 2B (778 Mb), DCH70-DCH80 detected earlier growth-rate associations relative to whole height. On chromosome 3B, the locus at 520 Mb (DCH90) was first identified through growth rate and only subsequently through whole height.

**Table 2:** Concordant marker-trait associations shared between ground canopy height and digital canopy model-derived traits in both populations.

Population	Chromosome	Marker	IWGSC_2.1 (Mb)
elite_panel	1A	Tdurum_contig61492_458	17.325
elite_panel	1B	Tdurum_contig29484_628	21.385
elite_panel	1D	Excalibur_c55959_651	12.725
elite_panel	2A	wsnp_Ex_c25445_34710489	12.932
elite_panel	2A	Excalibur_c39207_880	749.037
elite_panel	2B	Kukri_c96357_134	778.246
elite_panel	3B	Tdurum_contig49753_191	26.738
elite_panel	3B	BobWhite_c23887_53	41.180
elite_panel	3B	BS00034257_51	520.787
elite_panel	4A	TA002694-1263	163.514
elite_panel	4A	Excalibur_c4325_1440	686.663
elite_panel	4B	wsnp_Ex_c25373_34639805	481.851
elite_panel	4B	IAAV5117	496.356
elite_panel	4B	wsnp_Ex_c4358_7854194	505.321
elite_panel	4D	TG0011b	19.190
elite_panel	4D	wsnp_Ex_c49005_53739745	308.833
elite_panel	5A	wsnp_CAP11_c951_572693	35.025
elite_panel	5A	Tdurum_contig10210_425	571.808
elite_panel	5A	wsnp_Ex_c2702_5013188	586.926
elite_panel	5A	Tdurum_contig50175_875	596.175
elite_panel	5A	wsnp_Ra_c1146_2307483	673.927
elite_panel	5A	BS00022299_51	681.535
elite_panel	5B	Tdurum_contig49841_618	39.200
elite_panel	5B	IACX5702	580.685
elite_panel	5B	Excalibur_c3165_730	588.280
elite_panel	5B	RFL_Contig4167_1164	654.747
elite_panel	6B	wsnp_Ex_c25505_34771897	162.331
elite_panel	7A	Kukri_c12683_1309	31.965
elite_panel	7A	Kukri_c39994_227	72.194
elite_panel	7A	IAAV2865	132.385
elite_panel	7A	BS00109319_51	621.366
elite_panel	7A	wsnp_Ex_c5177_9174930	629.636
elite_panel	7B	Kukri_c51296_438	570.449
RIL	3A	RAC875_c55313_89	60.385
RIL	4B	AX-94545917	28.608
RIL	5A	AX-94429192	582.685

This temporal offset reflects the greater sensitivity of growth-rate traits to instantaneous genetic effects: growth rate captures the derivative of the height trajectory and therefore responds immediately when a locus begins to influence elongation, whereas whole height integrates these effects cumulatively and requires a longer period before a statistically detectable shift in the overall height profile emerges.

A percentile-dependent pattern also emerged, supporting prediction (b) from Section 2.3: the two lower mapping percentiles (DCH70-DCH80) were more frequently associated with growth rate, and these associations tended to yield lower *p-values*, whereas the two upper mapping percentiles (DCH90-DCH99) were predominantly associated with whole-height traits. This pattern was exemplified by the *Ppd-A1* region on chromosome 2A, where the MTA BS00036767_51 was confirmed by digital height values (JD_c3930_358), and by the *Vrn-A1* cluster on 5A (∼589 Mb), where growth-rate MTAs were predominantly detected at the DCH70-DCH80.

### 3.3 Sequential detection of major developmental loci after the tillering plateau

Temporal mapping revealed a striking developmental structure in the pattern of genetic associations (Fig. 4). In both populations, significant associations were detected during early seedling establishment (approximately 200-300 GDD), followed by a complete absence of significant associations during the tillering phase (approximately 600-700 GDD in the elite panel and 400-800 GDD in the RIL population), before associations re-emerged strongly during stem elongation and heading (approximately 700-2000 GDD in the elite panel; 800-2000 GDD in the RIL population).

This tillering plateau is physiologically coherent: during BBCH 20-29, vertical elongation is largely paused while resources are directed toward axillary bud initiation, tiller formation, and root system expansion [50]. Consequently, genotype-driven height differences cannot be phenotypically expressed during this phase, and genetic associations are undetectable regardless of the underlying transcriptional activity of the genes involved. The robustness of these findings was evaluated through a sensitivity analysis of the minimum persistence threshold (Section 2.6). At the 25-GDD threshold, the number of retained associations increased by approximately 70% relative to the reference, with most additional signals representing short-lived, non-recurrent associations that did not overlap with known developmental loci and were not replicated across years or populations, consistent with transient statistical noise. At the 100-GDD threshold, the number of retained associations decreased by approximately 20-25%, but all major loci (*Rht-B1, Rht-D1, Vrn-A1, Vrn-B1, Ppd-A1*) and the overall temporal detection sequence were fully preserved. At the more stringent 200-GDD threshold, only the longest-lasting associations were retained, resulting in a further reduction in the number of detected signals while maintaining the principal developmental regions and the tillering plateau. The tillering plateau was therefore consistently identified across all four thresholds, indicating that this pattern was robust to the analytical resolution employed. The 50-GDD window was consequently retained as a balanced threshold, preserving the major biologically interpretable and recurrent signals while reducing the contribution of short-duration associations.

### 3.4 Recurrent loci across years co-localize with known developmental regions

To distinguish environmentally stable genetic effects from season-specific signals, we identified MTAs and QTLs that were detected in at least two of the three growing seasons. Nine MTAs showed cross-year consistency in the elite panel (Table 3). These included the *Rht-D1* region on 4D (4-26 Mb), with a strong signal for growth rate and a weaker signal for whole height; two clusters on 5A (570-600 Mb) overlapping with *Vrn-A1*; a marker on 5B (∼580 Mb) near *Vrn-B1*, associated with both whole height and growth rate in 2022 and 2023; and additional recurrent loci on chromosomes 1A (552 Mb), 1D (12 Mb), 2D (603 Mb), 4A (709 Mb), and 6B (162 Mb) (Table 3). In the RIL population, the major loci at *Rht-B1* (4B) and *Vrn-A1* (5A) showed consistent associations across 2022 and 2023, although the timing of their detection varied between years, with *Rht-B1* detected at earlier developmental stages in 2022 than in 2023. No significant overlapping signals were detected in the 2024 RIL data, which may reflect the lower and more variable heritability estimates observed for DCH in that season (Table S5), possibly attributable to the drier spring conditions recorded in 2024 (Fig. 2c) and their effect on canopy uniformity. In particular, the dry 2023-24 growing season markedly reduced canopy height and compressed its range of variation across the RIL lines. At the peak of the season, RIL canopy height (DCH90) averaged 0.38 m in 2024, against 0.73 m in 2022 and 0.81 m in 2023; the among-line range narrowed to 0.46 m in 2024, from 0.60 m in 2022 and 0.92 m in 2023; and broad-sense heritability fell to 0.17, compared with 0.91 and 0.74 in 2022 and 2023, respectively (Table S5). Because QTL detection depends on the amount of heritable variation segregating among the lines, this compression of the height range - together with the lower heritability - reduced mapping power and is a major contributor to the reduced number of loci detected in the 2024 RIL population, without implying a drought-induced suppression of specific QTLs.

**Table 3:** Marker-trait associations consistently detected across growing seasons.

Population	Chromosome	Marker	IWGSC_2.1 (Mb)
elite_panel	1A	BS00030043_51	552.337
elite_panel	1D	RFL_Contig1338_2062	12.023
elite_panel	2D	tplb0021c10_951	603.068
elite_panel	4A	Tdurum_contig93100_149	709.77
elite_panel	4D	TG0011b	19.19
elite_panel	5A	TG0019	589.272
elite_panel	5A	TG0041	590.395
elite_panel	5B	IACX5702	580.685
elite_panel	6B	wsnp_Ex_c25505_34771897	162.331
RIL	4B	AX-94545917	28.608
RIL	5A	AX-94429192	582.685

Phenological phase-based comparison showed that the 2023-2024 season received substantially less rainfall during the vegetative and reproductive phases, with 75.9 and 14.9 mm, respectively, compared with 163.8 and 126.3 mm in 2022-2023. Median H^2^ across DCH70-DCH99 in the RIL population was also lower in 2024, reaching 0.14 during the vegetative phase and 0.28 during the reproductive phase. Only three non-redundant RIL QTLs, located on chromosomes 3A, 3B, and 7D, were detected during stem elongation in 2024, compared with 13 and 16 in 2022 and 2023, respectively, whereas 37 QTLs were detected after heading. However, the elite panel, which experienced the same environmental conditions, retained 30 non-redundant MTAs during stem elongation despite a similarly low median H^2^ of 0.26. Although MTA and QTL counts are not directly comparable, this contrasting temporal response indicates that the reduced RIL detection cannot be attributed solely to the drier conditions.

Across populations, the recurrent loci converged on five major chromosomal regions harbouring known adaptation genes: 2A (*Ppd-A1*), 4B (*Rht-B1*), 4D (*Rht-D1*), 5A (*Vrn-A1*), and 5B (*Vrn-B1*). The repeated detection of these loci across years, populations, trait types, and canopy percentiles strongly supports their biological relevance and indicates that the UAV-based temporal phenotyping platform captures genetically meaningful variation with high reproducibility.

### 3.5 Candidate gene prioritization highlights developmental regulators

Candidate gene analysis was performed using a two-step strategy (see Section 2.7). From all significant association intervals, QTG-Miner returned 163 prioritized genes, of which 98 were transcription factors (TFs; Table S15; Fig. S4). The most represented TF families were *WRKY* (the most abundant), *bHLH*, *ERF*, *HSF*, *C2H2*, and *RAV*.

Among the 87 non-redundant TFs associated with temporal MTAs/QTLs, the most biologically compelling candidates included the *DELLA Rht-D1* (*TraesCS4D03G0067100*) on chromosome 4D, accompanied by a *phosphatidylinositol-4-phosphate 5-kinase*, a *RuvB-like helicase*, and a *GeBP/storekeeper-like* transcriptional regulator within the same interval - all detected at late developmental stages consistent with the role of DELLA-mediated gibberellin repression in internode elongation. A diverse set of regulatory genes was found on chromosome 4B, including *TSO1-like*, *MYB-related*, *HD-ZIP*, *DOF*, *bHLH*, and *GRAS*, as well as ethylene-responsive factors and a B-type response regulator involved in two-component signalling, suggesting a complex developmental regulatory hub integrating meristem identity, hormone signalling, and stress-responsive transcriptional control. Early-stage temporal MTAs on chromosome 2A were enriched for *WRKY*, *B3*, and *bZIP*, pointing to early transcriptional reprogramming during canopy establishment, whereas a transcriptionally rich interval supported by both MTA and QTL analyses was found on chromosome 5A, containing *HSP*, *bHLH*, *ADA2-like* co-activators, and *WRKY* near the *Vrn-A1* region.

Seventy-four prioritized genes were associated with across-year concordant MTAs/QTLs (Table S15). Notable candidates in this set included *Rht-D1*, a *GRF5-like* gr*owth-regulating factor* on 4A, an *Early Heading Date 2-like C2H2 zinc finger* on 6B, and a *bHLH* annotated as a phytochrome-interacting factor (*PIF*) on 5A. Additional concordant candidates comprised TFs annotated as regulators of meristem or shoot development (orthologues of *RAX2*, *TB1*, *HOX7*, *TSO1*, and *WRKY1*), auxin-responsive genes (*IAA12*, *IAA13*, *IAA30*), and cytokinin-related response regulators (ORR41).

Twenty-four genes were instead shared between the temporally dynamic and the across-year concordant association sets (Table S15). This shared set included the DELLA gene *Rht-D1*, three *WRKY*s, one *TB1-like* branching repressors, *GeBP*-family transcription factors, three *RAVs* on chromosome 1A, and structural/cellular components (KIN-12G kinesins, PI4P5K phosphoinositide kinases, RuvB-like helicases). These 24 genes, repeatedly detected across years, developmental stages, and both mapping populations, represent a recurrently prioritized set of candidate genes for the genetic control of canopy height dynamics.

## 4 Discussion

### 4.1 UAV-based canopy height profiling provides robust and temporally resolved phenotyping

Our results confirm that UAV-derived digital canopy height faithfully reproduces ground-truth measurements and provides the temporal continuity required for the genetic dissection of growth dynamics. The strong agreement between DCH and GCH across percentile levels (R^2^ = 0.89–0.98; RMSE = 0.07–0.20 m) is consistent with - and in several cases exceeds - values reported in recent UAV-based wheat studies [51,52], and comparable to those obtained with LiDAR-based approaches in other cereal crops [18,25]. The percentile-based extraction approach proved particularly effective in minimizing noise from soil and low vegetation layers, with the highest percentiles exhibiting the lowest RMSE values. The finding that intermediate percentiles (DCH70-DCH90) show higher heritability than the DCH99 has practical implications for trait extraction in breeding pipelines, as these intermediate metrics capture the bulk canopy surface more reliably than extreme values influenced by individual awns, flag-leaf tips, apices of dominant tillers, or point-cloud reconstruction artifacts.

Importantly, the strong agreement between GCH and DCH should be interpreted not as a validation of the whole canopy maximum, but as a validation of the dominant canopy surface - the load-bearing layer measured by the cloth method, which corresponds empirically to the 80th–90th digital percentile. This is arguably the more genetically and agronomically informative metric, as it reflects collective canopy architecture rather than extreme outlier structures. This distinction has practical implications for the standardization of UAV-based phenotyping protocols: studies aiming to reproduce manual height measurements should preferentially extract the intermediate canopy percentiles (DCH70-DCH90) rather than the DCH99.

Taken together, these results confirm that UAV-based phenotyping now offers the temporal resolution, measurement stability, and throughput required to integrate dynamic plant architecture traits into genomic studies at the breeding scale.

### 4.2 Growth-rate traits provide superior temporal resolution for genetic mapping

The earlier detection of MTAs by growth-rate traits relative to whole-height traits is consistent with findings in sorghum [18], maize [53], and wheat [54]. Growth rate captures the instantaneous rate of change in canopy architecture and is therefore more sensitive to the onset of genetic effects on elongation - functioning as the temporal derivative of the height trajectory, whereas whole height reflects the cumulative integration of these effects over time. This complementarity argues for the routine inclusion of both trait types in temporal genetic studies of growth-related phenotypes.

A similar complementarity between whole-height and growth-rate traits has been reported in maize, where temporal GWAS identified stage-specific loci and showed that growth-rate traits can reveal associations not evident from terminal height alone [23]. Our results extend this concept to bread wheat and further show that the vertical layer sampled within the canopy influences the type of signal recovered, with lower percentiles more often associated with growth rate and upper percentiles more often associated with whole height. The consistency of the main patterns across alternative temporal windows (25, 50, and 100 GDD) indicates that these signals are robust to the analytical resolution adopted here. The independent adoption of the same 50-GDD temporal resolution by Sweet et al. (2024) [23] in maize provides cross-species support for this threshold as a biologically meaningful window for temporal genetic mapping in cereals. In their study, GWAS performed at 50-GDD intervals across the growing season revealed a total of 717 non-redundant SNPs associated with temporal plant height and growth rate, a figure comparable in magnitude to the 427 associations detected in our wheat populations; although direct numerical comparisons should be interpreted cautiously given differences in panel size, marker density, and significance thresholds, the similarity in order of magnitude suggests that this temporal scale captures a broadly similar density of genetic effects in both species despite their distinct growth habits and developmental architectures.

### 4.3 The tillering plateau: a physiological pause with implications for genetic mapping

The tillering plateau was one of the most consistent patterns in the temporal analysis. This pattern has not been previously reported in UAV-based wheat GWAS studies, despite being expected from standard phenological descriptions [55,56]. This was probably due to the fact that most prior investigations either lacked the temporal resolution to resolve this window or did not perform association mapping at sufficiently fine time intervals. Its consistent identification across years, populations, percentiles, and persistence thresholds (Section 2.6) confirms that it reflects a genuine developmental plateau rather than a statistical artifact. Importantly, the absence of phenotypic associations does not imply the absence of genetic activity: transcriptomic evidence indicates that *Vrn1* continues to accumulate during tillering [57,58], even though its consequences on canopy height are not yet manifest.

It should also be noted that RGB-derived canopy height samples only the vertical dimension of the canopy. During tillering, growth is mainly lateral, involving the production of new tillers, ground cover and early biomass, which do not change canopy height. The plateau reported here should therefore be read as a trait-specific pause in vertical growth rather than as an absence of genetic activity: canopy height is largely uninformative about tillering, which is better captured by canopy cover, tiller counts or spectral vigour indices [59,60]. Resolving the genetic control of this phase would require such complementary traits in addition to vertical height.

This finding has two important implications. First, the temporal boundaries of the tillering plateau provide an indirect estimate of the duration of the tillering plateau, a phenological parameter that is notoriously difficult to quantify manually in large breeding programs. UAV-derived canopy height trajectories could therefore serve as a proxy for tillering duration, enabling its routine estimation across hundreds of genotypes. Second, genotypic differences in the timing of exit from this plateau, rather than differences in early height per se, may condition when adaptation genes become phenotypically detectable, shaping downstream height trajectories.

In maize, Sweet et al. [23] noted that the variance explained by genotype was lowest early in the season and increased progressively, a trend consistent with a period of reduced genetic signal during early vegetative growth, but they did not report a discrete interval devoid of associations. This difference likely reflects the contrasting developmental programs of the two species: in wheat, vertical growth is temporarily suspended during axillary meristem activation, creating a prolonged tillering phase in which the main stem does not elongate; maize, by contrast, initiates internode elongation without an analogous pause, so that genetic effects on height accumulate continuously from early vegetative stages. The discrete plateau, therefore, appears to be a wheat-specific, or, more broadly, small-grain cereal-specific phenomenon rooted in the tillering growth habit. This observation underscores the value of crop-specific temporal phenotyping: although the general principle that different genes control height at different stages is shared across cereals [22,23,25], the precise temporal structure of genetic control is shaped by species-specific developmental programs that can only be revealed through sufficiently fine-grained temporal resolution.

### 4.4 Sequential detection of loci reveals a temporal hierarchy of developmental control

After the tillering phase, marker–trait associations re-emerged in a temporally structured manner in both populations. We interpret this pattern as a temporal ordering of locus detectability rather than as direct evidence of sequential gene activation [61,62]. In this framework, loci become detectable when their effects on canopy height are sufficiently expressed in the relevant developmental context, which may or may not coincide with the timing of their transcriptional activity.

In the elite panel (Fig. 4a), the earliest growth-rate associations were detected at approximately 390 GDD on chromosome 5B (∼580 Mb), near *Vrn-B1*. Additional early signals appeared shortly thereafter (410-500 GDD), involving loci on 7B, 7A, 5A (*Vrn-A1*), 2A (*Ppd-A1*), and 1A. Most of these signals persisted up to approximately 700 GDD; however, between approximately 700 and 820 GDD, the only growth-rate association remaining was the 5B locus, which thus bridged the vegetative and reproductive phases. After approximately 820 GDD, growth-rate associations became restricted to the 4D locus (∼19 Mb), tagging *Rht-D1*. This sequence is consistent with the established role of adaptation genes (*Vrn* and *Ppd*) in integrating thermal time and photoperiod cues to control the vegetative-to-reproductive transition [63], while *Rht* genes dominate later stages coinciding with internode elongation and gibberellin-mediated growth repression.

For whole-height associations, a parallel but temporally delayed pattern was observed: most signals clustered around 500-900 GDD, whereas the 4D locus was predominantly detected at later stages (1080–1600 GDD), confirming that growth rate captures the onset of genetic effects before they become visible in the cumulative height trajectory.

In the RIL population (Fig. 4b), the end of the tillering plateau was first marked by strong and recurrent associations at *Rht-B1* on chromosome 4B, followed shortly by signals at *Vrn-A1* on 5A. This order is consistent with the contrasting growth habits of the parental lines Lankaodali (spring type) and Rebelde (winter type), which segregate for key alleles at *Rht* and major developmental genes [27,30].

Notably, the temporal order in which loci became detectable differed between the two populations. In the elite panel, adaptation genes (*Vrn* and *Ppd*) were detected before *Rht*, whereas in the RIL population, *Rht-B1* appeared first, followed by *Vrn-A1*. This reversal reflects the distinct genetic architectures of the two panels: the elite panel comprises fixed cultivars with limited variation at *Rht* loci, allowing adaptation genes to emerge as the primary source of temporal variation; the RIL population segregates for major-effect *Rht-B1* alleles that produce strong architectural effects detectable immediately upon resumption of elongation. This comparison illustrates how UAV-based temporal phenotyping uncovers population-specific activation patterns that would remain entirely masked in conventional endpoint height measurements.

Overall, the temporal GWAS/QTL landscape delineates a coherent hierarchy: early canopy variation arises from regulators of meristem stability, epidermal differentiation, and metabolic allocation; mid-season dynamics involve adaptation genes (*Vrn* and *Ppd*) mediating the vegetative-to-reproductive transition, and late-stage growth is associated with GA-responsive repressors (*Rht*) and their downstream targets.

This temporal ordering is conceptually consistent with reports from other cereals, although the identity and sequence of the loci differ among species. Such differences likely reflect species-specific developmental programs and highlight the importance of interpreting temporal associations in the context of crop biology rather than assuming a universal developmental sequence. In maize, Adak et al. [22] reported that QTLs controlling early-season growth involved brassinosteroid biosynthesis (*brd1*) and auxin transport (*pin11*) genes, whereas later QTLs were associated with flowering-time regulators such as *zcn8* and *rap2.7*. This temporal sequence represents the inverse pattern we observed in wheat, where adaptation genes were detected before gibberellin-related *Rht* genes, reflecting differences in developmental architecture: maize lacks a vernalization requirement and a prolonged tillering phase, so the primary axis of temporal genetic variation relates to hormonal control of internode elongation rather than to the vegetative-to-reproductive transition. In wheat, the sequential fulfilment of vernalization and photoperiod requirements creates a temporal structure in which adaptation genes necessarily precede structural growth genes.

### 4.5 Biological interpretation of temporal association patterns considering known expression profiles of adaptation genes

The temporal association patterns observed for the major loci are broadly consistent with the known developmental roles of *Vrn*, *Ppd*, and *Rht* genes [49]. Here, the comparison with published expression and functional studies is not intended as formal validation, but rather as a biological framework for interpreting why these loci become phenotypically detectable at different stages of canopy development.

#### 4.5.1 Vernalization genes (*Vrn-1*): stage-dependent expression consistent with pre- and post-tillering association signals

In winter wheat, *Vrn-1* expression is epigenetically repressed during early vegetative growth and is progressively induced by prolonged cold exposure [58,64]. Studies on TILLING mutants demonstrated that *Vrn-1* regulates transcript levels of the *Vrn-2* repressor in leaves, maintaining them at low levels after vernalization to allow production of the mobile FT protein and flowering initiation [65]. Crucially, *Vrn-1* and its paralogues *FUL2* and *FUL3* have redundant functions not only in floral transition but also in stem elongation, as demonstrated through double and triple null mutants in tetraploid wheat [66]. Furthermore, allelic variation at *Vrn-A1* has quantifiable pleiotropic effects on stature: the *Vrn-A1c* allele confers shorter stature compared with *Vrn-A1b* or the recessive *Vrn-A1*, with differences of approximately 5-10 cm [13,14,67], indicating that these loci influence canopy architecture through genuine structural remodeling rather than merely indirect effects of altered flowering time.

In our dataset, associations at the *Vrn-A1* locus on 5A exhibited a biphasic pattern: a brief activation during late emergence/early tillering (winter) followed by reactivation during stem elongation. This profile is consistent with the transcriptional dynamics reported by Murai et al. [68] and Kitagawa et al. [57], who showed that *Vrn-1* expression levels increase gradually during the vegetative phase even under non-vernalizing conditions in controlled environments, with a marked acceleration at the vegetative-to-reproductive transition. Using near-isogenic lines, Emtseva et al. [69] confirmed that *Vrn-B1* alleles specifically influence the duration of the tillering phase, reinforcing the interpretation that the tillering plateau observed in our study corresponds to a genuine physiological pause during which *Vrn-1* expression is accumulating but has not yet reached levels sufficient to produce measurable phenotypic effects on canopy height.

In the RIL population, the *Vrn-A1* signal emerged later than in the elite panel and was specifically associated with the vegetative-to-reproductive transition. This timing is consistent with the allelic contrast between the parental lines Lankaodali (spring type) and Rebelde (winter type) [27,70]: lines inheriting the winter *Vrn-A1* allele transitioned later to stem elongation, resulting in a delayed rise in daily growth rate during the tillering phase that our UAV phenotyping system precisely captured. Thus, in this population, *Vrn-A1* primarily affects the timing and intensity of stem elongation rather than early vegetative growth.

#### 4.5.2 Photoperiod sensitivity genes (*Ppd-1*): effects on pre-anthesis phase duration

The *Ppd-1* genes, located on chromosome group 2, encode pseudo-response regulators that modulate the photoperiod response [70]. Beyond developmental timing, photoperiod-insensitive alleles exert well-documented pleiotropic effects on plant stature: *Ppd-D1a* is associated with height reductions of approximately 10-15% and *Ppd-B1a* with reductions of 5-13%, largely attributable to the shortened duration available for internode extension [71,72]. Detailed studies using near-isogenic lines demonstrated that photoperiod-insensitive alleles (*Ppd-D1a*) reduce the duration of the emergence-to-onset of stem elongation phase in a less-than-additive manner [73], while after the onset of stem elongation, sensitivity to current photoperiod decreases markedly and is no longer correlated with *Ppd-1* variability. Bloomfield et al. [12] showed that photoperiod sensitivity at *Ppd-D1* lengthens the stem elongation phase by up to 23%, and that this effect depends on *Vrn-1* allelic composition and vernalization status. Furthermore, Gauley et al. [74] recently demonstrated that *Ppd-1* regulates the developing inflorescence transcriptome, influencing stage-specific expression of genes involved in auxin signalling and meristem identity. In our study, associations at the *Ppd-A1* locus on 2A appeared during early stages (∼200-500 GDD), consistent with the role of *Ppd-1* in integrating environmental signals during canopy establishment. The effect of *Ppd-D1* on the regulation of phenological phase duration [75] supports the interpretation that genotypic differences in the timing of exit from the tillering plateau are partly conditioned by variation at *Ppd-1*.

#### 4.5.3 Reduced height genes (*Rht-1*): constitutive expression but stage-specific phenotypic effect

Unlike *Vrn* and *Ppd* genes, the *Rht-B1* and *Rht-D1* genes encoding DELLA proteins are expressed relatively constitutively across all stem tissues [76]. Pearce et al. [76] demonstrated through qRT-PCR and promoter-GUS constructs that the three homoeologs *Rht-A1*, *Rht-B1*, and *Rht-D1* are expressed at comparable levels across different regions of the elongating stem, with slightly higher levels in the peduncle and lower portion of the first internode; the tissues are undergoing active cell expansion. This pattern is consistent with our finding: associations at *Rht* loci were detected by our UAV system only when internode elongation begins, not because the genes are “switched on” at that point, but because DELLA-mediated growth repression becomes phenotypically detectable only when the biological substrate (elongation) is active. *Rht* genes thus provide the clearest illustration of the principle introduced in Section 4.4: constitutive expression can produce stage-specific phenotypic effects when the relevant developmental substrate is temporally restricted.

In the RIL population, *Rht-B1* was detected even at the beginning of tillering, a finding that is physiologically consistent with the GA-insensitive *Rht-B1b* allele's known effect on coleoptile length and early shoot growth [77]. High-resolution DCH data captured this early architectural effect, which precedes the stronger influence of *Rht-B1* during stem elongation and further illustrates the temporal layering of genetic control within a single locus.

Comparative transcriptomic studies between mutant and wild-type *Rht-B1* alleles further identified that *DELLA* proteins regulate downstream expression of genes involved in cell expansion (AGPs, arabinogalactan proteins) and cell wall lipid biosynthesis in a tissue- and stage-specific manner [78]. The recent identification of *PLATZ-A1* (*RHT25*) as a height regulator that genetically interacts with *Rht1* and is maximally expressed in the stem during early elongation stages (BBCH 30 - BBCH 32) [79] provides further support for the view that temporal height regulation involves a network of interactions between DELLA repressors and other stage-specific transcription factors.

### 4.6 Candidate genes reveal regulatory modules associated with canopy height across developmental stages

The candidate-gene analysis extends the major-locus results by highlighting additional recurrent candidates whose annotated functions are compatible with the developmental regulation of canopy growth. These candidates should be interpreted as prioritized hypotheses rather than as validated causal genes.

During the early vegetative phase (200-500 GDD), temporal signals on chromosome 2A were enriched for *AP2/ERF*, consistent with regulatory programs linking stress responsiveness and developmental control in plants [80]. Within the same interval, the presence of a *WUSCHEL-related homeobox* (*WOX*) aligns with the established role of *WOX* proteins in meristem maintenance and vascular organization [81], supporting the view that fine-tuning meristematic activity establishes the architectural baseline before internode expansion begins.

At later stages (∼700-2000 GDD), temporal MTAs/QTLs converged on canonical regulators of height and architecture. Strong and repeated signals for *Rht-B1* and *Rht-D1* confirm the central role of DELLA-mediated gibberellin suppression in controlling internode elongation and determining the semi-dwarf phenotype. The parallel detection of *TB1*-like genes, recognized as repressors of axillary growth and modifiers of inflorescence architecture [82,83], indicates that branching-control pathways continue to influence canopy height even after tillering, likely through effects on resource allocation and main-stem dominance.

The 24 genes shared between temporal and across-year concordant MTAs/QTLs define a robust regulatory backbone. At its centre lie the *DELLA* genes *Rht-B1* and *Rht-D1*, whose recurrent detection at multiple developmental stages reflects their well-known role in modulating gibberellin sensitivity. The presence of *TB1*-like transcription factors within this stable gene set highlights the contribution of branching-control pathways, while additional TF families, including *GeBP*-family and *G2-like* involved in nutrient signalling and cytokinin–GA interactions, *NAM-like NACs* regulating senescence and nutrient remobilization [84], and *DOF* and *ZF-HD* associated with vascular patterning and meristem identity, underscore a broader regulatory network integrating meristem function, nutrient signalling, and hormonal crosstalk. Beyond transcriptional regulators, the shared set includes structural and cellular components such as *KIN-12G* kinesins, *PI4P5K* phosphoinositide kinases, and *RuvB*-like helicases, pointing to roles for intracellular dynamics, vesicle trafficking, and chromatin remodeling in shaping growth trajectories [85].

Collectively, these candidates delineate a multilayered regulatory system integrating GA signalling (*DELLAs*), branching repression (*TB1*-like TFs), meristem and vascular stability (*WOX, DOF, ZF-HD*), nutrient remobilization (*NAM-like NACs*), and cellular architecture (kinesins, phosphoinositide kinases, helicases). The consistency of this core across environments, populations, and developmental stages suggests that it represents a conserved axis of genetic control over canopy height dynamics. From a breeding perspective, these genes constitute promising targets for manipulating the timing and rate of growth transitions or stabilizing plant architecture under variable environmental conditions.

### 4.7 Limitations and future directions

The two mapping populations were of moderate size (174 elite cultivars and 162 RILs) and were genotyped with array-based platforms that, after filtering, retained relatively low marker densities (9805 and 3746 SNPs, respectively), with especially sparse and less polymorphic coverage of the D subgenome, which was correspondingly underrepresented in our results (e.g., a single QTL on chromosome 6D). Consequently, statistical power for small-effect loci was limited, and minor-effect and D-genome associations were likely under-ascertained. Denser genotyping (higher-density arrays, exome capture, or whole-genome sequencing), larger panels, and multi-environment trials will be required to recover small-effect and D-genome loci and to refine the relatively broad candidate intervals identified here toward causal genes.

In addition, it should be noted that co-localization within ± 2 Mb does not constitute evidence of causation. The candidate genes identified here represent biologically plausible candidates that require functional validation through approaches such as TILLING mutant analysis, gene editing, or detailed expression studies before causal relationships can be established.

Indeed, a natural extension of this work will be to resolve the major loci at the allelic level, for instance, by reconstructing multi-marker haplotypes to compare allele-specific growth trajectories and by relating the candidate intervals to validated functional variants, such as the diagnostic *Rht-B1*, *Ppd-1*, and *Vrn-1* polymorphisms. Such analyses are best pursued with denser or diagnostic genotyping and larger panels, which would allow the prioritized candidates to be examined at the level of individual alleles and functional variants.

## 5 Conclusions

This multi-season UAV-based study demonstrates that temporal canopy height profiles derived from RGB imagery provide a robust and biologically informative framework for dissecting the genetic architecture of wheat development. The key findings are as follows: (i) growth-rate traits detect genetic associations earlier than whole-height traits, functioning as more sensitive indicators of instantaneous genetic effects on elongation; (ii) an apparent tillering plateau separates early and late phases in which developmental loci become detectable, providing an indirect estimate of a phenological parameter that is otherwise difficult to quantify at the breeding scale; (iii) adaptation genes (*Vrn* and *Ppd*) dominate the vegetative-to-reproductive transition, while *Rht* genes are associated with late-season height, and the temporal order in which they became detectable differs between populations in a manner consistent with their allelic architecture; (iv) comparison with published transcriptional profiles is consistent with these temporal patterns, distinguishing genes with constitutive expression but stage-specific phenotypic effects (*Rht*) from genes with stage-dependent expression (i.e., *Vrn* and *Ppd*); and (v) a recurrently prioritized set of 24 candidate genes, including *DELLAs*, *TB1*-like branching repressors, and regulators of meristem function and nutrient signalling, is proposed as candidate regulators of canopy height trajectories across seasons and populations.

From a genomic perspective, the integration of temporal phenotypic profiles with genomic prediction models represents a promising avenue for breeding applications. Future work coupling multi- and hyperspectral sensors with genomic prediction models that incorporate growth-curve parameters, such as inflection points, maximum growth rates, and asymptotic heights, could substantially improve selection accuracy for dynamic traits by capturing developmental trajectories rather than single-time-point snapshots.

Nonetheless, the present results demonstrate that digital canopy height is not merely a structural trait but a dynamic phenotype encoding the timing, rate, and coordination of key developmental transitions, and support the integration of temporal UAV phenotyping into breeding programs targeting growth timing, plant architecture, and environmental adaptation. More broadly, this study establishes a general framework, applicable to other traits and crops, for integrating high-throughput temporal phenotyping with public transcriptional resources, bridging the gap between association genomics and functional genomics.

## Author contributions

P.D.V., S.E., D.*P. and* F.F. wrote the manuscript with input from all the authors. F.F., I.*P. and* P.S. conducted the field trials and phenotyping under the supervision of P.D.V. F.F. developed the code for digital trait estimation and conducted the statistical analysis. F.F. and D.P. performed the genetic analysis. G.A., I.C. and S.E. investigated the candidate genes. All authors read and approved the final manuscript.

## Data availability

The complete code and data that support the findings of this study are available from the corresponding author (P. D. V.) upon reasonable request due to ongoing collaborative work.

## Funding

This work was supported by the Italian Ministry of Enterprises and Made in Italy - MIMIT in the framework of a project entitled “Sistemi avanzati di modellizzazione digitale per il miglioramento e la predizione di resa e qualità nella produzione cerealicola italiana” - GRANO.IT - Nr. F/360091/01-02/X75 (CUP B19J24000140005).

## Declaration of competing interest

The authors declare that they have no known competing financial interests or personal relationships that could have appeared to influence the work reported in this paper.

## Footnotes

Appendix B Supplementary data to this article can be found online at https://doi.org/10.1016/j.plaphe.2026.100279.
