# Supplementary material for: Temporal GWAS and QTL mapping of UAV-derived digital canopy height reveals stage-specific genetic effects in bread wheat
Source: Plant Phenomics. 2026 Sep 3;8(4):100279. doi: 10.1016/j.plaphe.2026.100279 (PMC13582003; doi:10.1016/j.plaphe.2026.100279)
Supplement: Supplementary file 1 [file mmc2.pdf]

Temporal GWAS and QTL mapping of UAV-derived canopy height reveals sequential activation of developmental genes in bread wheat.

Fabio Fania[1,2], Patrizio Spadanuda[1,2], Damiano Puglisi[1], Giuseppina Angione[1,2], Ida Colella[1,3], Ivano Pecorella[1], Salvatore Esposito[4], Pasquale De Vita[1]

[1] CREA Research Centre for Cereal and Industrial Crops, CREA-CI, Foggia, Italy

[2] Department of Agriculture, Food, Natural Resources and Engineering (DAFNE), University of Foggia, Foggia, Italy

[3] Department of Agriculture and Forest Sciences, University of Tuscia, 01100 Viterbo, Italy

[4] CNR-IBBR, National Research Council of Italy, Institute of Biosciences and Bioresources, Portici (NA), Italy

Corresponding Author: Pasquale De Vita, pasquale.devita@crea.gov.it

Supplementary figures

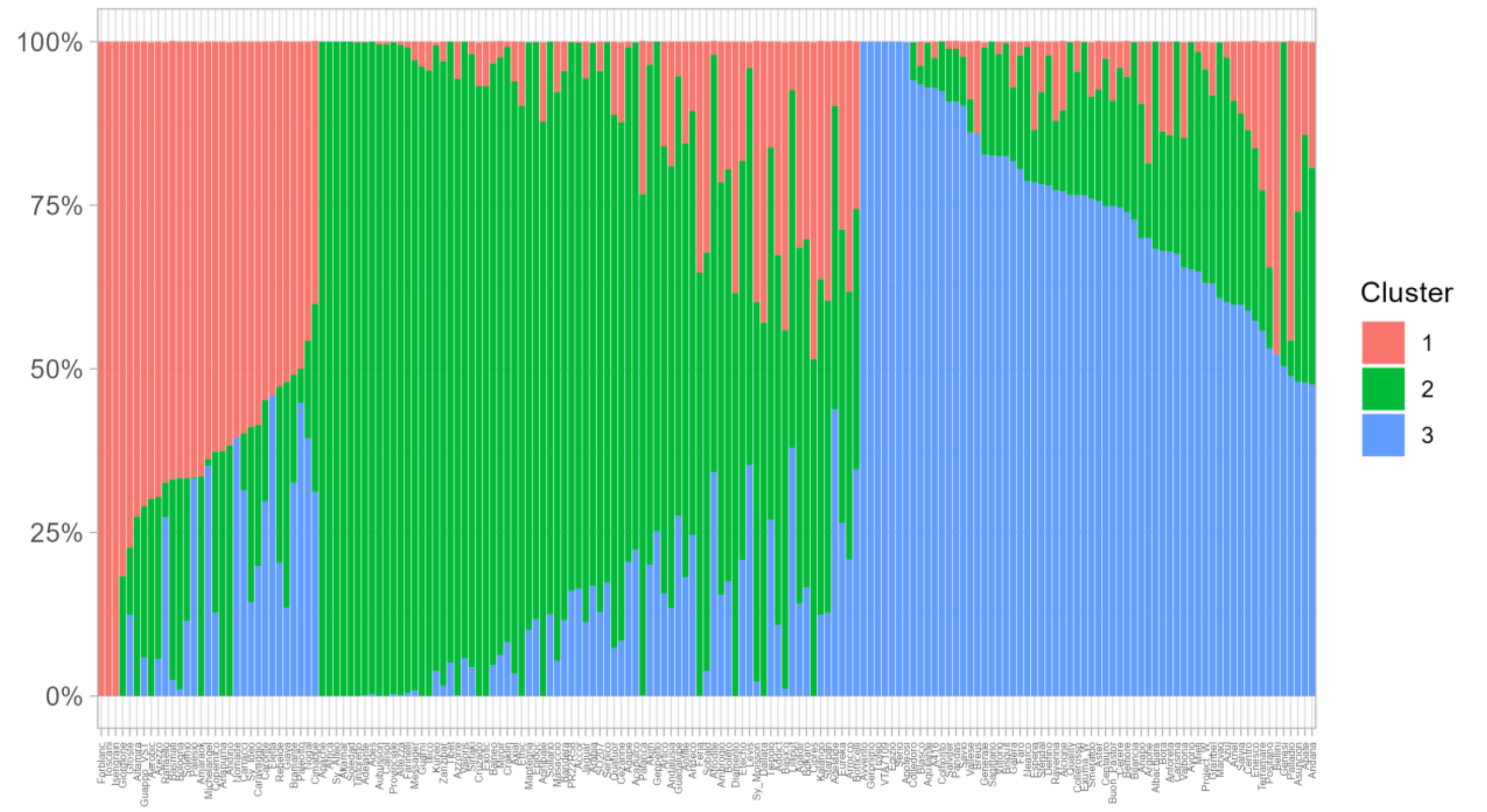

Fig. S1 Population structure of the elite panel inferred using STRUCTURE at K = 3. Each vertical bar corresponds to one genotype, and the colored segments represent the estimated membership proportions (Q values) of each individual in the three inferred genetic clusters.

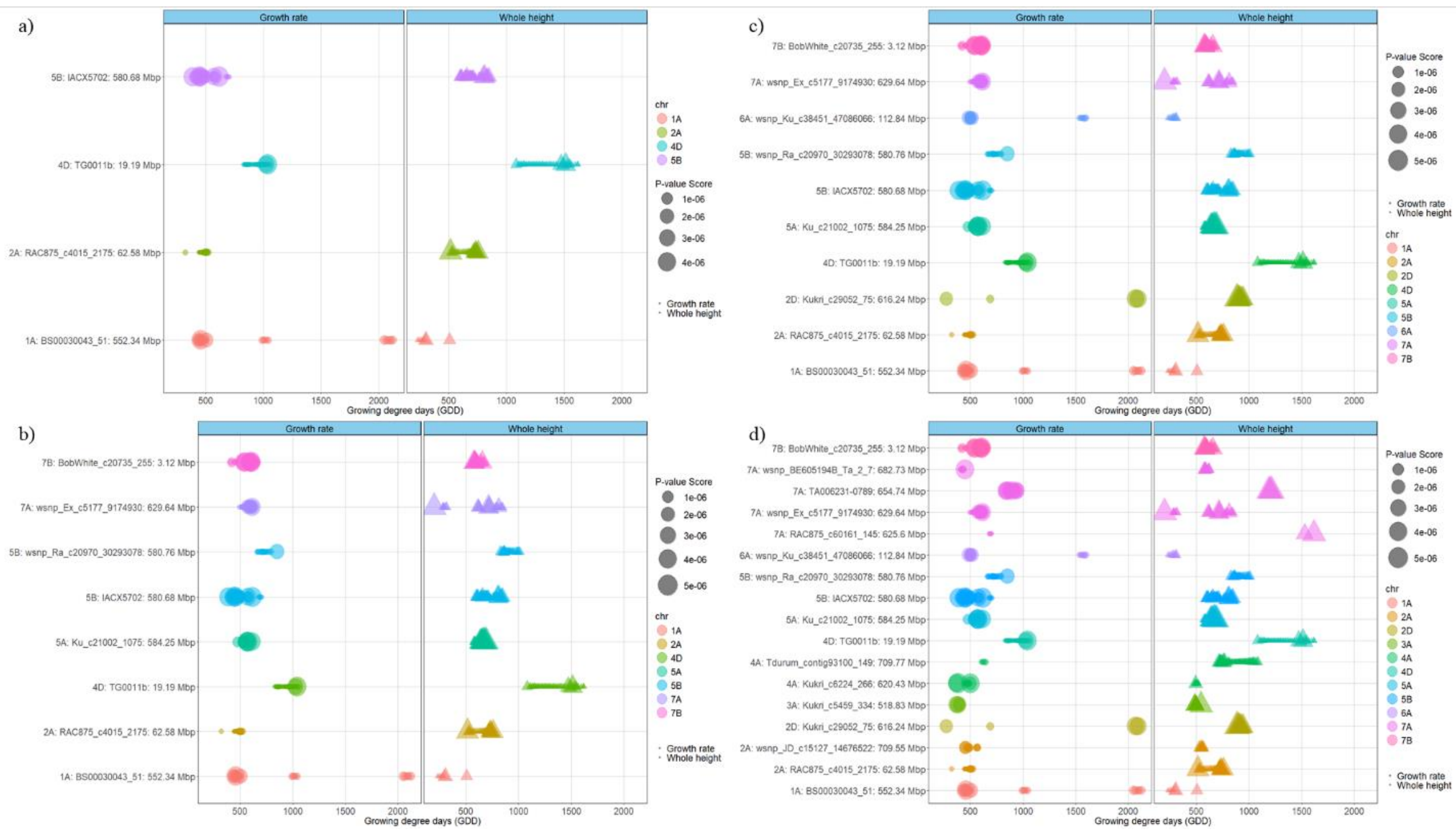

Fig S2. Sensitivity analysis of temporal association windows in the elite panel under different GDD thresholds: a) 200, b) 100, c) 50, and d) 25 GDD. To evaluate the robustness of the temporal association analysis to window size, retained associations were compared across thresholds in terms of their chromosomal distribution and temporal boundaries. For each panel, results are shown separately for growth rate and whole height. Colors indicate chromosomes, whereas point size reflects the association significance score. The 50-GDD threshold was used as the reference setting for the main text.

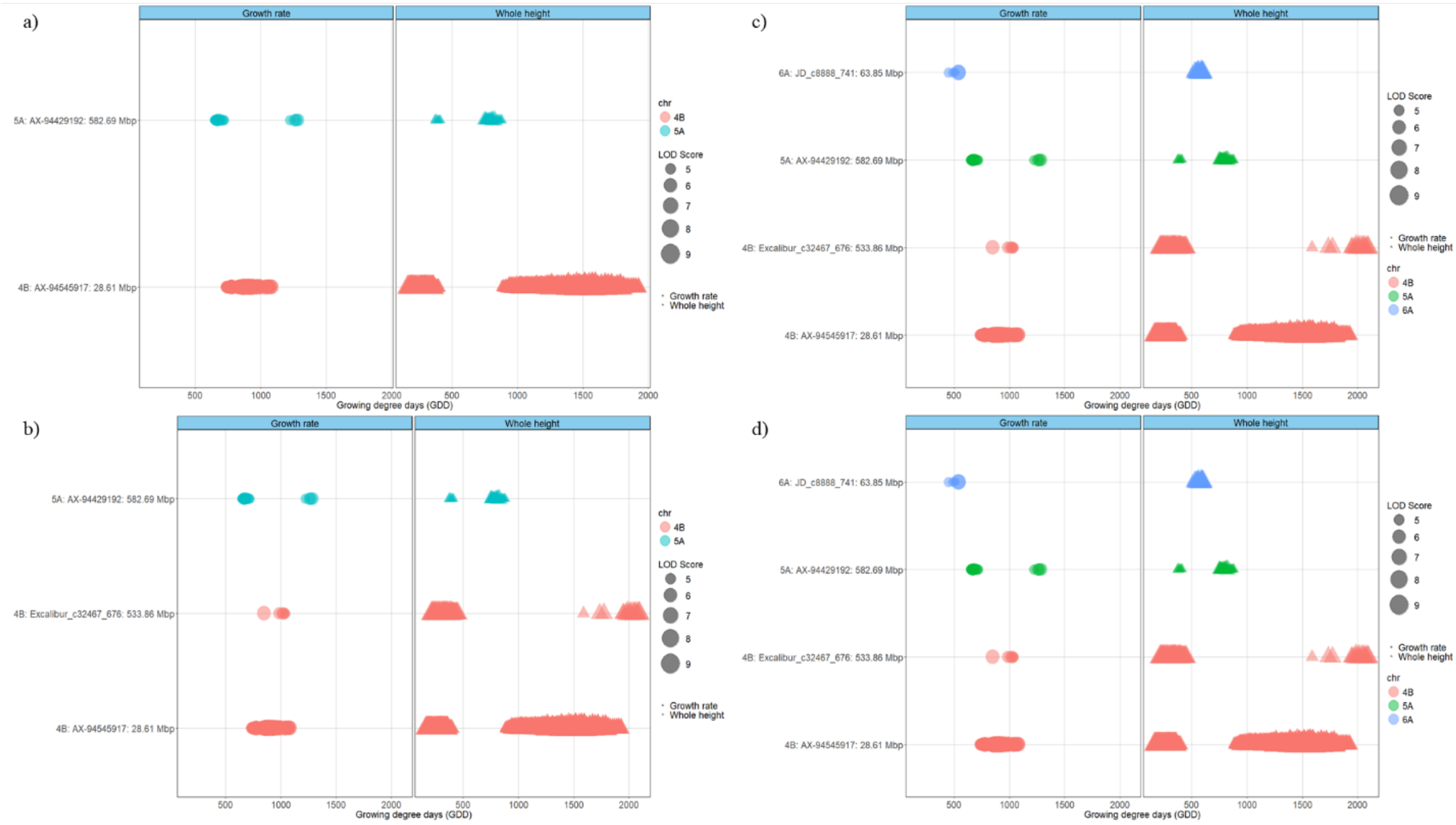

Fig S3. Sensitivity analysis of temporal association windows in the RIL population under different GDD thresholds: a) 200, b) 100, c) 50, and d) 25 GDD. To evaluate the robustness of the temporal association analysis to window size, retained associations were compared across thresholds in terms of their chromosomal distribution and temporal boundaries. For each panel, results are shown separately for growth rate and whole height. Colors indicate chromosomes, whereas point size reflects the association significance score. The 50-GDD threshold was used as the reference setting for the main text.

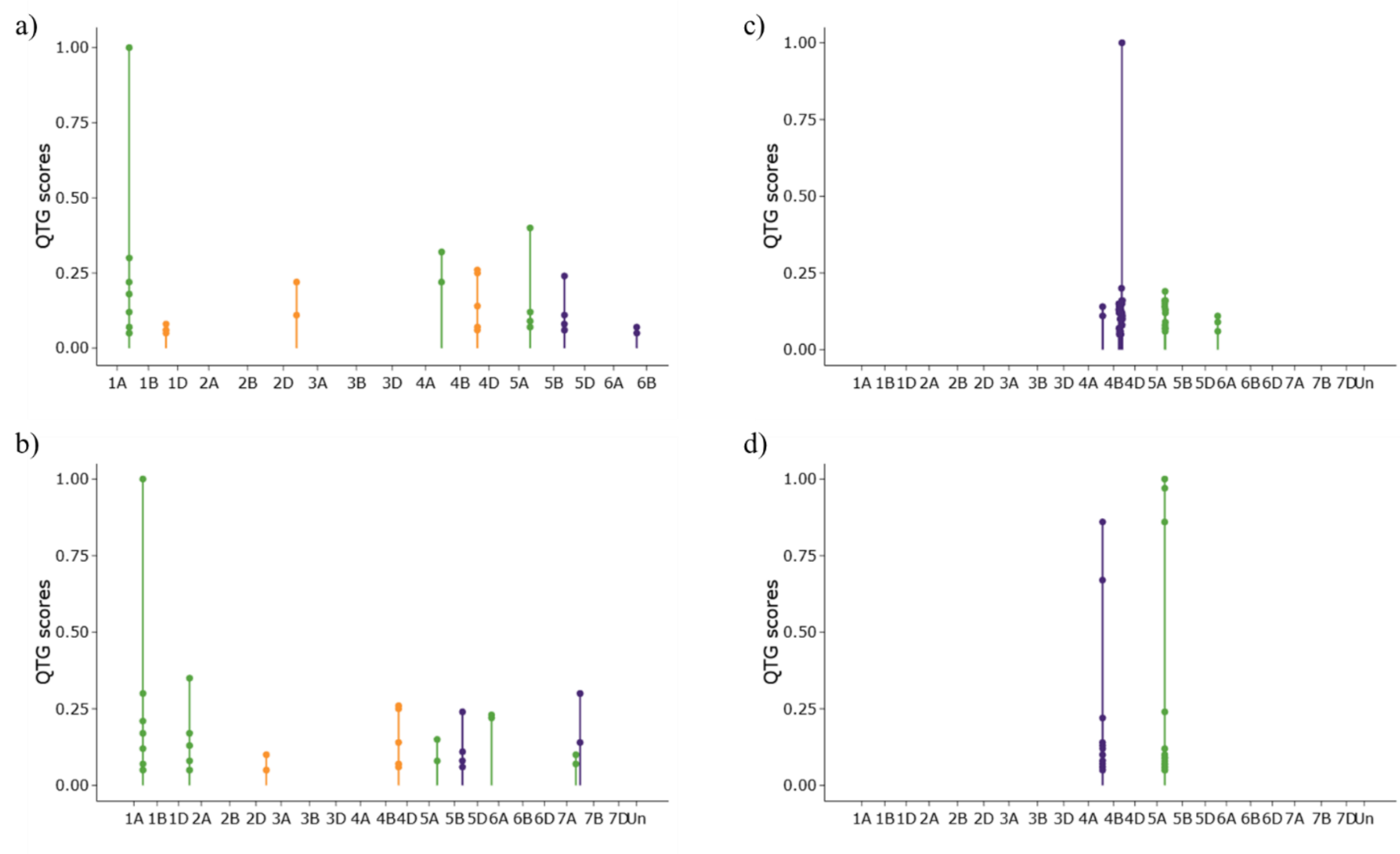

Fig S4. QTG-Miner prioritization results for candidate genes identified in the RIL population and elite panel. Panels show a) concordant signals in the RIL population, b) temporal signals in the RIL population, c) concordant signals in the elite panel, and d) temporal signals in the elite panel.

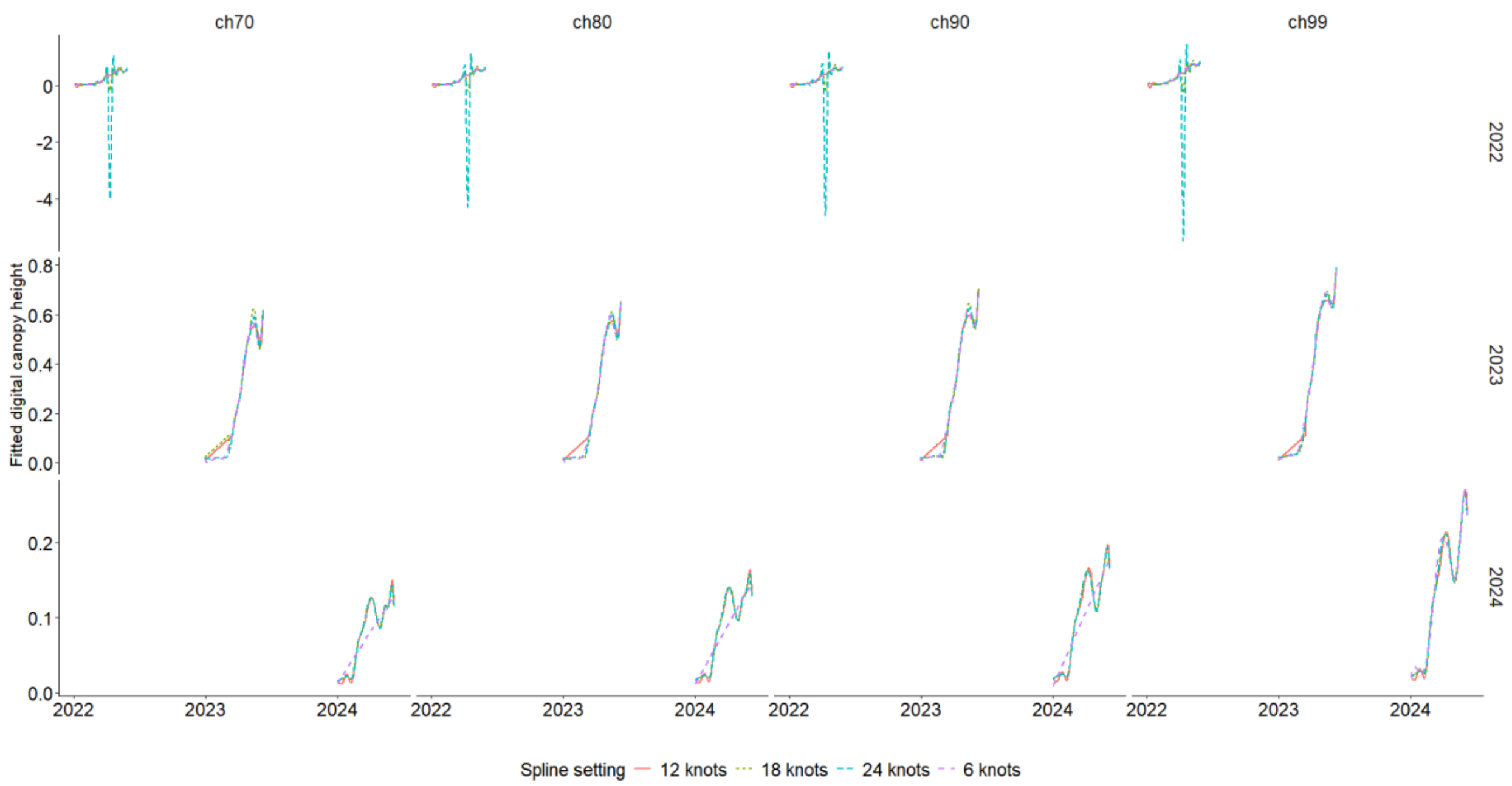

Fig S5. Sensitivity analysis of P-spline fitting for the representative check genotype Mec. Fitted digital canopy-height trajectories for the 70th, 80th, 90th,

and 99th percentiles (ch70–ch99) were compared across the 2022, 2023, and 2024 growing seasons using 6, 12, 18, and 24 knots.
